# Structure-Based Search for New Inhibitors of Cholinesterases

**DOI:** 10.3390/ijms14035608

**Published:** 2013-03-11

**Authors:** Marek Bajda, Anna Więckowska, Michalina Hebda, Natalia Guzior, Christoph A. Sotriffer, Barbara Malawska

**Affiliations:** 1 Department of Physicochemical Drug Analysis, Faculty of Pharmacy, Medical College, Jagiellonian University, Kraków 30-688, Medyczna 9, Poland; E-Mails: marek.bajda@uj.edu.pl (M.B.); anna.wieckowska@uj.edu.pl (A.W.); michalina.ignasik@uj.edu.pl (M.H.); natalia.guzior@uj.edu.pl (N.G.); 2 Institute of Pharmacy and Food Chemistry, University of Wuerzburg, Wuerzburg D-97074, Am Hubland, Germany; E-Mail: sotriffer@uni-wuerzburg.de

**Keywords:** acetylcholinesterase, butyrylcholinesterase, cholinesterases inhibitors, drug design, fragment-based design

## Abstract

Cholinesterases are important biological targets responsible for regulation of cholinergic transmission, and their inhibitors are used for the treatment of Alzheimer’s disease. To design new cholinesterase inhibitors, of different structure-based design strategies was followed, including the modification of compounds from a previously developed library and a fragment-based design approach. This led to the selection of heterodimeric structures as potential inhibitors. Synthesis and biological evaluation of selected candidates confirmed that the designed compounds were acetylcholinesterase inhibitors with IC_50_ values in the mid-nanomolar to low micromolar range, and some of them were also butyrylcholinesterase inhibitors.

Abbreviations

AChE: acetylcholinesterase
AD: Alzheimer’s disease
BuChE: butyrylcholinesterase
hBuChE: human butyrylcholinesterase
NMDA: *N*-methyl-D-aspartate
PAS: peripheral anionic site
PDB: Protein Data Bank
TcAChE: *Torpedo californica* acetylcholinesterase

## 1. Introduction

**Figure 1:**
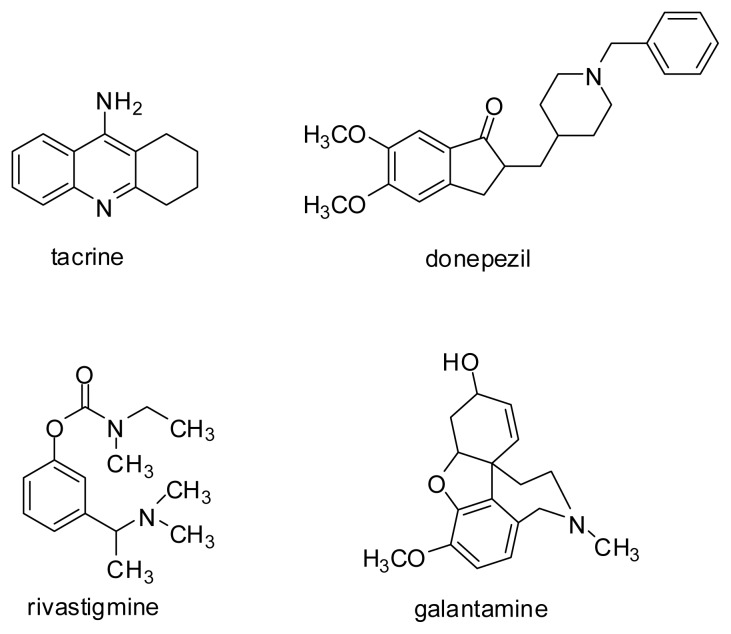
Cholinesterases inhibitors used in Alzheimer’s disease (AD) treatment. Tacrine was withdrawn, due to its hepatotoxicity, but it is still used in experiments as a reference inhibitor.

Since the cholinergic hypothesis was approved, cholinesterases have become important therapeutic targets in Alzheimer’s disease (AD) treatment [1]. The mechanism of action of cholinesterase inhibitors comprises the increase of cholinergic transmission in the central nervous system, defects of which lead to memory and thinking disturbances. Nevertheless, this kind of treatment is not fully effective and the search for new drugs involves different therapeutic possibilities. Currently, only drugs from the cholinesterases inhibitors group, such as rivastigmine, donepezil and galantamine, and from the *N*-methyl-D-aspartate (NMDA) antagonist group, such as memantine, have been approved (Figure 1) [2,3]. During the last few years, it has been shown that cholinesterases can influence a series of other processes, such as β-amyloid aggregation, due to the presence of a peripheral anionic site (PAS) in their structure [4,5].

These discoveries led to a resurgence of interest in cholinesterases as an important target in the therapy of Alzheimer’s disease, and numerous research groups have undertaken investigations concerning design and synthesis of new inhibitors [6,7].

Cholinesterases differ in substrate specificity and susceptibility to various kinds of inhibitors [8,9]. Due to these differences, they were divided into two types: acetylcholinesterase (AChE), which hydrolyzes acetylcholine, and butyrylcholinesterase (BuChE), which is able to hydrolyze larger molecules, such as butyrylcholine. Both cholinesterases belong to a large protein family containing the α/β hydrolase fold [10,11]. Acetylcholinesterase is the main enzyme metabolizing acetylcholine. It is also responsible for cerebral blood flow modulation, β-amyloid aggregation, activation and expression of APP95 protein, τ protein phosphorylation and has an influence on inflammatory processes. It interacts with β-amyloid, leading to creation of stable complexes and formation of senile plaques [12,13]. The other enzyme—butyrylcholinesterase—is also important, because of its ability to hydrolyze acetylcholine and other choline esters. It was observed that the BuChE level increases in AD patients. Its role is not fully understood, but some studies suggested that it could promote amyloid plaque formation, and therefore, the search for inhibitors of both enzymes has been undertaken [6,14].

The structure of AChE was first described in 1991 by J. Sussman, who successfully crystallized the enzyme from the electric organ of the electric ray, *Torpedo californica* (TcAChE) [15]. TcAChE is composed of 537 amino acids and contains 12 β-sheets surrounded by 14 α-helices. BuChE is very closely related to AChE; therefore, all structural analyses were performed with a homology model of BuChE based on electric ray acetylcholinesterase, until its structure was solved [16].

**Figure 2:**
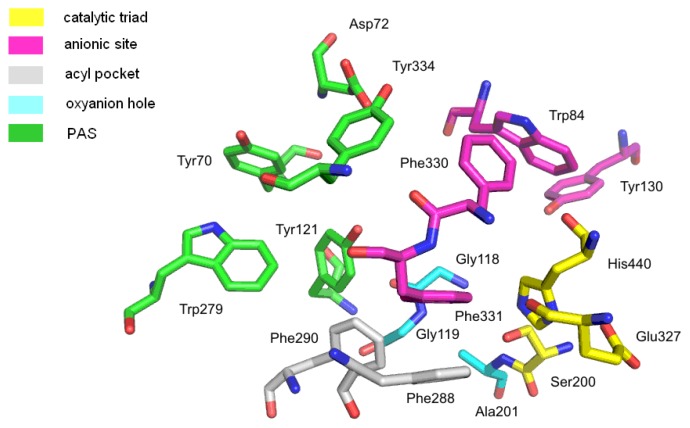
Active site of acetylcholinesterase (*Torpedo californica* acetylcholinesterase (TcAChE); Protein Database Bank (PDB): 1EVE).

**Figure 3:**
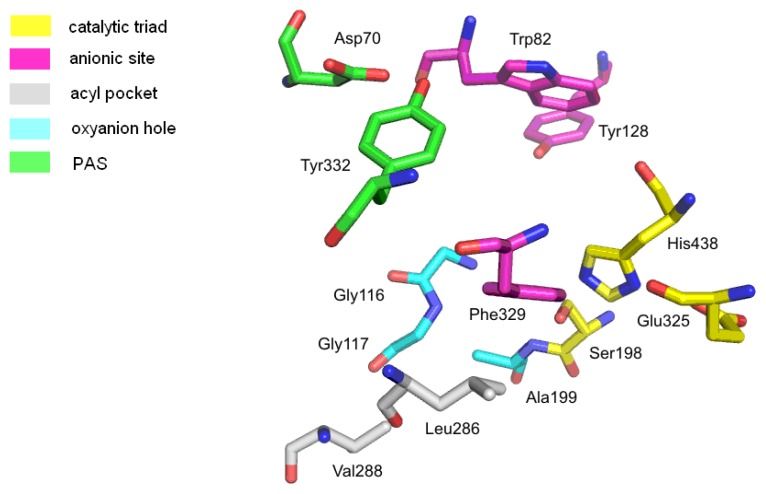
Active site of butyrylcholinesterase (human butyrylcholinesterase (hBuChE), PDB: 1P0I).

The hydrolysis reaction takes place in the catalytic site of AChE, located at the bottom of a deep, and narrow gorge (20 Å deep, 5 Å wide), which is composed of conserved aromatic amino acids (Figure 2). This most important site, called also the esteratic site, contains the three essential amino acids, Ser200, His440 and Glu327 (TcAChE), which create the catalytic triad. They are involved in the transfer of the acetyl group from acetylcholine to Ser200. The catalytic triad of human BuChE is built of Ser198, His438 and Glu325. An essential role in the hydrolysis process is also played by aromatic amino acids, such as Trp84 and Phe330 [17]. The AChE anionic site, composed of Trp84, Tyr130, Phe330 and Phe331, is responsible for binding the substrate quaternary ammonium group with cation-π interactions. Due to the interactions with the anionic site, the proper orientation of acetylcholine in the gorge is provided. It also enables inhibitor binding to the enzyme. In BuChE, the tryptophan key residue (Trp82) is conserved, but one phenylalanine (Phe330) is replaced by Ala328. The lack of this phenylalanine influences the affinity of some inhibitors. For example, huperzine is characterized by high affinity to AChE, because its protonated primary amine creates strong interactions with Phe330 from TcAChE, whereas affinity to hBuChE is lower, due to the presence of Ala328 in this place [18,19]. One of the serine hydrolase features is stabilization of the transition state by amino acids of the oxyanion hole through hydrogen-bond creation. During the enzymatic reaction, the transition complex is created and stabilized by Gly118, Gly119 and Ala201 from AChE. In hBuChE, the oxyanion hole is similar and consists of highly conserved N–H dipoles, derived from amino acids of the main chain: Gly116, Gly117 and Ala119. The acyl pocket is responsible for substrate specificity (Figure 3). Comparison of hBuChE and TcAChE shows differences in size, especially of the acyl binding pocket. The active gorge is larger in hBuChE than in TcAChE (500 Å^3^*versus* 300 Å^3^). The active site in TcAChE is lined by 14 aromatic residues [20], in BuChE, six of them are replaced by smaller aliphatic residues, even polar ones. The shape of the acyl pocket is determined by two residues: these are the aromatic amino acids Phe288 and Phe290 in TcAChE, but the aliphatic residues Leu286 and Val288 in hBuChE. Phe288 and Phe290 prevent access of larger molecules to the catalytic center. In the case of BuChE, the replacement of two phenylalanine residues by the smaller amino acids, valine and leucine, makes the hollow in the acyl pocket larger and enables larger molecules to get in, which results in lower enzyme specificity. The AChE peripheral anionic site (PAS) consists of five amino acids—Tyr70, Asp72, Tyr121, Trp279 and Tyr334. The PAS is located at the entry to the active gorge and is responsible for extra activities, including the interaction with β-amyloid [15,21]. BuChE does not own counterparts of Tyr70, Tyr121 and Trp279 in the peripheral site. The PAS is a very important structural element, responsible also for binding of many inhibitors.

These differences in the structure of the active sites can explain the majority of the activity differences of the two enzymes.

**Figure 4:**
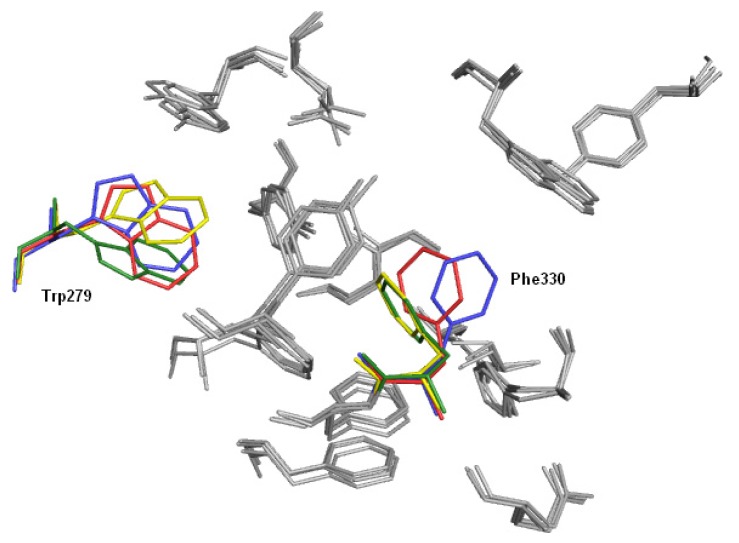
Superimposed amino acid residues from the AChE active site, derived from different crystal structures (1ACJ: yellow; 1EVE: blue; 2ACE: red; 2CKM: green).

Alignment of the crystal structures of native AChE and its complexes with inhibitors (tacrine THA, PDB: 1ACJ; decamethonium, DECA, PDB: 1ACL; m-(*N*,*N*,*N*-trimethylammonio)-trifluoroacetophenone, TMTFA, PDB: 1AMN; huperzine, HUP, PDB: 1VOT; edrophonium, EDR, PDB: 2ACK; donepezil, E2020, PDB: 1EVE) highlighted conformational changes of Phe330 and Trp279 upon ligand binding (Figure 4). In addition, the crucial role of conserved water molecules was revealed [22]. Phe330 can adopt a wide range of conformations in complexes with inhibitors. Based on its χ1 angle values, AChE crystal structures can be divided into three groups: group A—similar to native AChE: 2ACE (χ1 = −162°), 1AMN (χ1 = −174°), 1VOT (χ1 = −171°), 2ACK (χ1 = −177°); group B—similar to AChE in complex with tacrine: 1ACJ (χ1 = 157°); and group C—similar to AChE in complex with donepezil: 1EVE (χ1 = −130°), 1ACL (χ1 = −117°). Group A includes inhibitors, which bind near the bottom of the active gorge. In the case of tacrine (group B), the phenyl ring of Phe330 is placed over the ligand molecule and creates together with the indole moiety of Trp84 a characteristic sandwich with the inhibitor between them. Group C includes inhibitors extended along the active gorge, which causes location of Phe330 near the wall of the gorge. In the case of group B, there is also a different conformation of Trp279 in comparison to the native enzyme or AChE from other complexes (χ2 = 30° and 90°, respectively). This may suggest the presence of a special linkage between the anionic subsite and the peripheral anionic site. It is interesting to note that the side chains of other amino acids are relatively rigid in comparison to Phe330. The presence of this residue also leads to the increased catalytic activity of acetylcholinesterase relative to butyrylcholinesterase (which does not contain Phe330).

All crystal structures of complexes of AChE with inhibitors contain many water molecules. Among them, there are buried water molecules, which are closed inside restricted areas, and conserved waters, which are almost always present in the same position, regardless of the complex. Most important for the drug design process are conserved waters in the active site, because they are often involved in ligand binding. The analysis of five crystal structures of AChE complexes showed many water molecules, present in the active gorge and engaged in creation of hydrogen bonds [23]. There are 20 water molecules in the active site of AChE from 2ACE, 15 from 1VOT, 18 from 2ACK, 16 from 1EVE and 22 from 1VXR.

In the active center of BuChE, derived from four different complexes, 31 conserved water molecules were observed. They might be important, due to their ability to create interactions with ligands and enzymes by so-called water bridges.

The search for new active substances from the group of cholinesterases inhibitors has revealed various classes of chemical compounds. The literature presents several studies concerning the derivatives of drugs, such as physostigmine, rivastigmine, tacrine, donepezil, galantamine or huperzine [6,24]. Recent literature data presents very active, selective inhibitors towards both enzymes or non-selective inhibitors [25–27].

On the other hand, the research also involves multitarget directed ligands, *i.e.*, compounds that join anticholinesterase activity with beta-amyloid anti-aggregation properties, inhibition of monoamine oxidase, β-secretase, histamine H_3_ receptor antagonism, metal-complexing properties, voltage-dependent calcium channel antagonistic activity, serotonin reuptake inhibition or with antioxidant and neuroprotective properties [28,29]. Generally, such multifunctional ligands showed lower biological potency towards all targets in comparison with selective ligands. Among them, dual binding site cholinesterases inhibitors represent a valuable therapeutic strategy for further development of potential anti-AD agents [30,31].

**Figure 5:**
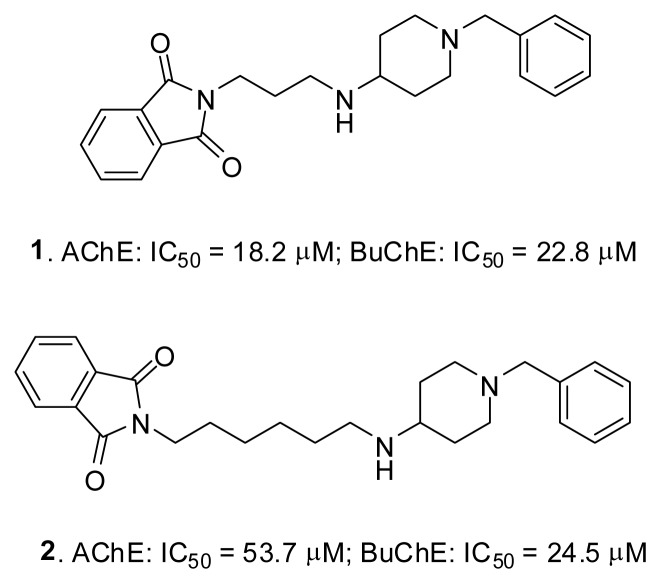
The starting point of our research—compounds **1** and **2**. They are both acetyl- and butyryl-cholinesterase inhibitors with micromolar IC_50_ values [32].

Our research group has been involved in the development of cholinesterases inhibitors as potential anti-AD drugs. The results of previous studies [32,33] were the starting point of this research. We have obtained different series of compounds, among which hybrid structures with an *N*-benzyl piperidine moiety were the most interesting, as they showed inhibition of both cholinesterases with activities in the micromolar range. Two of them are presented in Figure 5. Previous docking studies showed a high analogy in the context of interactions with cholinesterases. These compounds were dual binding site inhibitors. They were arranged along the gorge of acetylcholinesterase, creating characteristic π–π stacking interactions with Trp84 in the catalytic site by the benzyl substituent and with Trp279 at the peripheral anionic site by heteroaromatic moiety. They also formed a cation-π interaction with the Phe330 residue. In addition, there were other interactions stabilizing the inhibitor-AChE complex, such as hydrogen bonds involving water molecules. In the case of butyrylcholinesterase, the benzylpiperidine moiety interacted with Trp82 and the phthalimide fragment in the acyl pocket (Leu286, Val288) [32].

These results prompted us to design novel dual binding site cholinesterases inhibitors with improvement of inhibitory activities. Herein, we describe in detail the results of an extensive molecular modeling study for the design of cholinesterase inhibitors. For this purpose, the molecular modifications approach of our active compounds and fragment-based design have been applied.

## 2. Results and Discussion

### 2.1. Analysis of Enzyme Structures

#### 2.1.1. Acetylcholinesterase

The Protein Data Bank (PDB) contains 122 crystal structures of acetylcholinesterase complexed with different ligands [34]. From these complexes, 11 with the most popular ligands were selected. These are the complexes with the following ligands: tacrine, decamethonium, edrophonium, galantamine, donepezil, rivastigmine, huperzine A, acetylcholine (modeled into native structure), ganstigmine, bis-7-tacrine and pralidoxime (abbreviations are described in the caption to Table 1). They are characterized by acceptable resolution from 2.15 Å for bis-7-tacrine to 2.80 Å for edrophonium. Mutual complex comparison using the program PyMOL [35] showed that root-mean-square deviation (rmsd) values for all protein atoms are in the range from 0.702 Å to about 5 Å. Due to the key role of the amino acids contained in the active center, a detailed structural analysis of this part of the enzyme was conducted. The obtained rmsd values are summarized in Table 1.

The analysis confirmed the relatively high degree of structural conservation of amino acids in acetylcholinesterase and conformational changes of Phe330 and Trp279, which was mentioned in the literature [22].

#### 2.1.2. Butyrylcholinesterase

The PDB contains 31 structures of butyrylcholinesterase complexes with ligands [34]. Among them, four structures (1P0I, 1P0M, 1P0P, 1P0Q) were selected for further analysis. These were complexes with products of hydrolysis of the substrate, *i.e.*, butyrate and choline, and with the substrate analogue, *i.e.*, butyrylthiocholine after soman inhibition. They were characterized by good resolution within 2.0–2.43 Å. The selected structures were compared with PyMOL [35]. Rmsd values for all protein atoms were in the range 1.025–1.596 Å, which indicates, as in the case of AChE, a high similarity of the 3D structure of the enzymes from different complexes. It should also be noted that butyrylcholinesterase is devoid of the two amino acid residues corresponding to Phe330 and Trp279 in AChE, which were characterized by high conformational freedom. Analysis of BuChE complexes, taking into account the presence of water molecules, showed that there were 31 water molecules present in the active center of these four complexes. They were used during the docking of ligands to the active center of BuChE.

### 2.2. Validation of Docking with Gold Software

It was decided to use seven crystal complexes with the non-covalently bound inhibitors (1EVE, 1ACJ, 2CKM, 1DX6, 1ACL, 1AX9 and 1VOT) for redockings to acetylcholinesterase to optimize the parameters associated with the process of docking. The size of binding site, presence of water molecules and scoring function were taken into account. Table 2 summarizes the best settings for all redocking processes with Gold Software [36].

In the next step, cross-dockings were performed. Each inhibitor was docked to the enzyme from each complex, using settings from Table 2 with respect to the protein. The quality of fit was evaluated on the basis of ligand rmsd values (Table 3) with the following ranges:

- rmsd ≤ 1.0 Å, good pose;
- Å < rmsd ≤ 2.0 Å, close pose;
- Å < rmsd ≤ 3.0 Å, pose with errors;
- rmsd > 3.0 Å, bad pose.

The performed cross-dockings showed that the best structure of the enzyme that could adopt new AChE ligands was one from the complex 1EVE, to which it was possible to dock three inhibitors with rmsd in the range between 0.8 and 1.4 Å. The structure of acetylcholinesterase from the complex with bis-tacrine was also suitable. Other enzymes only allowed for a good docking of the ligand with which they were crystallized. In further studies, it was decided to use the structure of acetylcholinesterase from the 1EVE complex, due to the structural similarities of donepezil and the analyzed compounds, especially from our own library. The studies related to the search for new inhibitors were also designed to obtain new compounds capable of interaction with both the active site and the PAS. The choice of the 1EVE structure also seemed to be right due to this reason.

**Figure 6:**
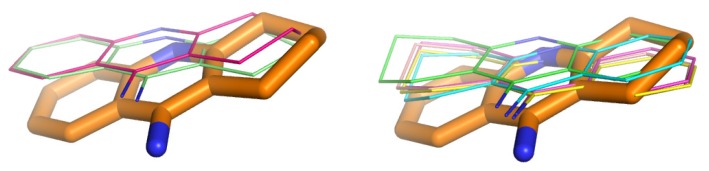
Tacrine clusters obtained in docking to BuChE. Results were compared with the arrangement of tacrine (orange) in the complex with acetylcholinesterase.

In the case of butyrylcholinesterase, the docking validation process was more difficult, because most of the available crystal structures were complexes with organophosphorus compounds, which irreversibly block the enzyme, due to the formation of a covalent bond. It was decided to modify the validation of the docking, using some information from the literature [8]. Structure 1P0I and tacrine, an inhibitor of both cholinesterases, were chosen as the basis for validation. In the PDB database, there is a crystal structure of AChE available with this inhibitor (1ACJ), so in the first stage of the study, the structure of both enzymes was compared. The superimposition of the two structures in the PyMOL program gave an rmsd value equal to 3.725 Å for all protein atoms. High similarity (73% aa) and identity (53% aa) suggested large similarities in the structure of the enzymes and the possibility of interaction between tacrine and BuChE in a similar way. Tacrine was docked into the enzyme, according to the method described in the Experimental section. There were two main clusters of solutions. One of them was almost identical to the arrangement of tacrine in the complex 1ACJ, and the second one contained the tetrahydroacridine moiety still in the same plane, but turned. The benzene and cyclohexene rings replaced each other. The top-ranked pose obtained a value of 52.63 for the ChemScore function. In both cases, the characteristic π–π stacking for tacrine and the residue of Trp82 was observed (Figure 6). Based on the obtained results, it was decided to use BuChE from 1P0I complex and the described settings for further studies.

### 2.3. Modifications of Heterodimeric Structures from Our Library

**Figure 7:**
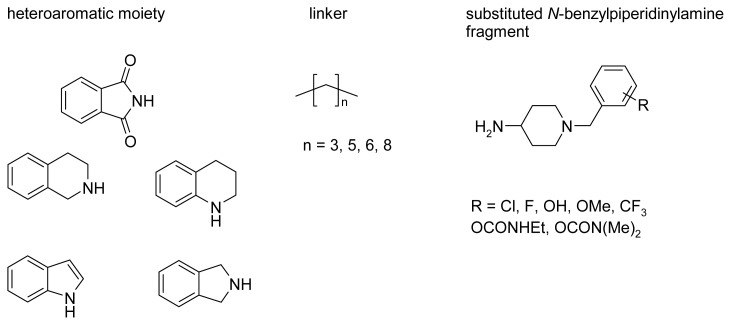
Modifications of heterodimeric compounds **1** and **2**.

Isoindoline-1,3-dione derivatives (Figure 5) were the basis for the initial design of novel inhibitor structures. To improve the activity, some modifications of heterodimeric molecules were introduced. They concerned all fragments of the molecule: the heteroaromatic moiety, the linker and the substituent in the *N*-benzylpiperidinylamine group. The structural changes of these compounds tested *in silico* are illustrated in Figure 7.

The structures of complexes obtained in the process of docking were accurately analyzed to find beneficial changes improving binding. The influence of all modifications on the binding was assessed by the ChemScore function and inspected visually.

The first step of the analysis included changes of the heteroaromatic moiety. Five heterocyclic systems were taken into account: phthalimide, indole, tetrahydroisoquinoline, tetrahydroquinoline and isoindoline. Initial investigations were carried out for the derivatives with a three-carbon linker, which was characteristic for compound **1**. It was shown that phthalimide had provided the most profitable interactions with acetylcholinesterase and had the highest score. It is worth noting that this moiety is similar to the indanone present in the donepezil structure and may be considered as a bioisostere. The benzylpiperidine moiety interacted with Trp84 by π–π stacking, the protonated piperidine created cation-π interactions with Phe330 and a hydrogen bond with a water molecule. The amine group interacted with Tyr334 (cation-π). The phthalimide created π–π stacking with Trp279 and CH-π interactions with Tyr70. This moiety was favored, because it formed two extra H-bonds by carbonyl groups: one with a water molecule and the other with the hydroxy group of Tyr121. The other heterocyclic moieties were assessed lower. The same analysis was performed for the derivatives of compound **2** with a six-carbon tether. In this case, all heterocyclic systems had a similar score. It was possible, because the longer chain made the phthalimide slightly shift and its carbonyl groups formed much weaker hydrogen bonds.

The second step of the analysis included further changes of tether length (four and eight carbon atoms). Compounds with four methylene groups and different heterocyclic moieties were similarly assessed as the derivatives with a three-carbon chain. In case of compounds with the longest linker (8-CH_2_–), the differences were enhanced to the advantage of three moieties: isoindoline, tetrahydroquinoline and indole. It might be caused by π–π stacking of these moieties with Trp279, which was more profitable than the interactions formed by phthalimide.

The third stage of the analysis included the influence of substituents in the *N-*benzylpiperidine moiety on the activity. The presence of halogen atoms, hydroxy, methoxy, trifluoromethyl and carbamate groups in different positions was evaluated. Docking results showed that the enzyme, which has a relatively limited space within the gorge, might accept only the compounds with small substituents, such as halogens or hydroxyl group. Compounds with hydroxy group, fluorine and chlorine showed similar binding modes. All interactions described earlier were kept; the hydroxy group and fluorine gave an extra H-bond. Larger substituents forced molecules to make a small shift such that the *N*-benzylpiperidine could not create similarly beneficial interactions with Trp84 and Phe330. It turned out that the optimal position of the hydroxy group is a *para* substitution, because it was particularly preferred to the creation of a hydrogen bond with Glu199. For the fluorine atom the preferred position was *ortho*, due to interaction with Ser200, and for the chlorine atom *meta*.

According to this analysis, the following compounds were selected as potential AChE inhibitors: *p*-hydroxy, *m*-chloro and *o*-fluoro derivatives of compounds **1** and **2** and similar substituted analogues with isoindoline, tetrahydroquinoline or indole and eight-carbon linker.

Analysis of the binding modes of the selected compounds with butyrylcholinesterase suggested that only the introduction of the same substituent (OH, Cl, F) in the *N*-benzylpiperidinyl moiety could slightly improve activity, whereas other structural changes could not change the activity to a large extent.

**Figure 8:**
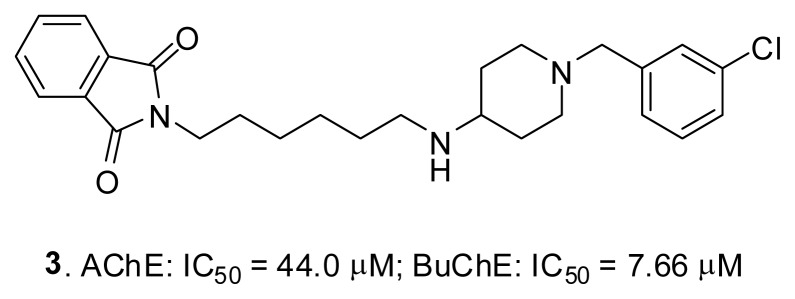

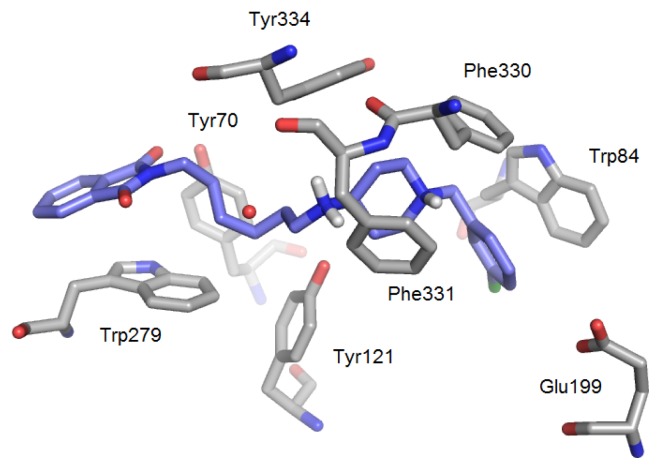
The obtained derivative **3**, based on our library of compounds.

One of the designed structures, compound **3** (Figure 8), was synthesized and tested in Ellman’s assay [37]. It corresponds to the *m*-chloro derivative of compound **2**. The small structural modification of a chlorine atom in the *meta* position increased the activity against both targets: in the case of AChE, from 53.7 to 44.0 μM, and in the case of BuChE, from 24.5 to 7.66 μM.

### 2.4. Fragment-Based Design of Cholinesterase Inhibitors

**Figure 9:**
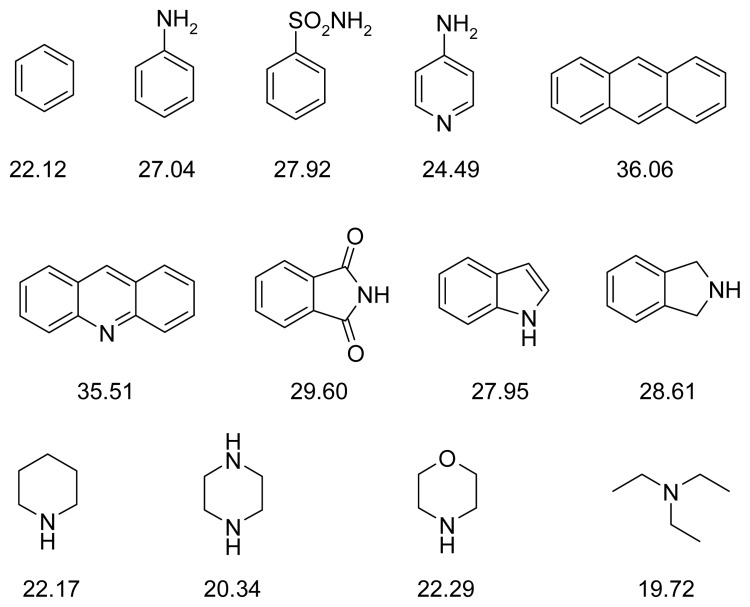
Structural fragments used for the fragment-based design of cholinesterase inhibitors. ChemScore values for binding with acetylcholinesterase are given for each fragment.

According to the fragment-based methodology [38], a group of structurally simple fragments was chosen (Figure 9), and areas where they created optimal interactions with the enzyme were identified by means of the docking procedure.

In the case of AChE for the aromatic compounds, π–π stacking and hydrophobic interactions with Phe330, Phe331 and Tyr334 residues and, in some instances, with Trp84 or Trp279, were found. Piperidine, piperazine and morpholine formed cation-π interactions with these residues. Additionally, these heterocyclic rings could create hydrogen bonds with Asp72 and His440. Acridine, aniline and triethylamine formed a hydrogen bond with Tyr121, but benzenesulfonamide with Tyr121, Tyr334 and Asp72.

With reference to BuChE, the aromatic compounds formed π–π stacking and hydrophobic interactions mainly with Trp82, Trp430 or Trp231. Hydrogen bonds with Asp70 and Tyr332 for aniline and benzenesulfonamide were also observed. Piperidine, piperazine, morpholine and triethylamine formed cation-π interactions with Trp82 and Trp430; cyclic amines also created hydrogen bonds with Tyr332 and, in some cases, additionally with Asp70.

**Figure 10:**
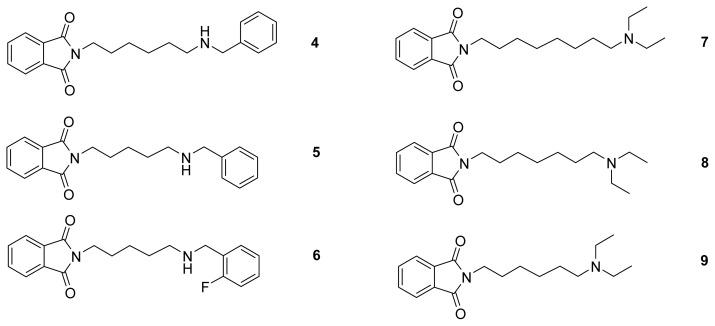
Structures of designed acetylcholinesterase inhibitors—newly synthesized (**4**–**6**) and currently described compounds (**7**–**9**).

Based on these fragments, some hybrid structures were designed (Figure 10). Each heterodimer contained two fragments, connected by a different kind of tether. The most popular fragment was phthalimide. It was present in most compounds (**4**–**9**), because it created, except for π–π stacking, two H-bonds in the PAS of AChE, which made it a preferable moiety. The peripheral anionic site of BuChE is smaller, which explains why compounds bound poorly with that enzyme. The second fragment interacted with the catalytic active site (CAS). Ligands **4** and **5** included a benzylamine. This change increased the basicity in comparison with other amines and improved cation-π interactions with Phe330. The best tether length for those compounds was five to six. Compound **6** was a simple modification of compound **5**. It contained a fluorine atom in the benzyl moiety in the *ortho* position. Due to this substituent, an extra interaction with the hydroxy group of Ser200 was formed. Compounds **7**–**9** included triethylamine as the second fragment. One of the ethyl substituents in triethylamine was incorporated in the linker. The tether length was in the range of six to eight, with the optimum equaling eight. Protonated amine gave cation-π interactions with Trp84 and Phe330. The assessment of novel ligands was based on the docking procedure. The obtained ligand-enzyme complexes were analyzed visually and evaluated using the ChemScore function (Table 4). At the step of validation, it was shown that compounds with IC_50_ (AChE) < 1 μM obtained a ChemScore higher than 40; therefore, ligands for which the scoring function reached that value (compounds **4**–**8**; exception: **9**—almost 40) were selected for synthesis. Synthesis and biological evaluation of inhibitors **7**–**9** have been presented recently [39].

All designed compounds were arranged along the gorge of acetylcholinesterase, interacting in a similar manner. They created interactions with main amino acids, Trp84, Phe330 and Trp279, and the hydrogen bond with a water molecule.

In the case of butyrylcholinesterase, compounds **4**–**9** were arranged in a similar way, but their conformations were slightly bent, not linear, as for AChE. The fit of the new compounds was not as good as in the case of AChE, suggesting that these compounds might inhibit acetylcholinesterase stronger than butyrylcholinesterase. The structures interacted with two main amino acids Trp82 and Trp231. They formed a characteristic π–π and cation-π interactions. The linkers between the outermost elements of the molecule were involved in the hydrophobic interactions with the aromatic ring of Tyr332.

Six compounds, which were designed according to the fragments, were synthesized and tested in Ellman’s assay [37]. They showed selective acetylcholinesterase inhibitory activity with micro- and submicromolar IC_50_ values, confirming the expectations based on the molecular modeling results. The most active was compound **6**, with IC_50_ = 87 nM. This was 2-(5-(2-fluorobenzylamino)pentyl)isoindoline-1,3-dione, a heterodimeric structure composed of *o*-fluorobenzylamine, phthalimide and a five-carbon chain. The inhibitor showed a binding mode with an extended conformation. The benzyl moiety created CH-π interactions with Trp84. The protonated amine group created cation-π interactions with Phe330. It was also engaged in an H-bond network: amine group-water molecule-Tyr121. The tether was located in the middle of the active gorge, near the aromatic amino acids, Phe290, Phe331 and Tyr334, where it creates hydrophobic interactions. The phthalimide moiety forms π–π stacking with Trp279 and CH-π interactions with Tyr70. Both carbonyl groups are engaged in H-bonds: one with Tyr121 and the other with water. The fluorine atom gave an additional H-bond with Ser200, which increased the activity in comparison with the unsubstituted compound **5** (87 nM *vs*. 170 nM). Also, the elongation of the linker (compound **4**, IC_50_ = 330 nM) slightly decreased the activity, because in this case, the fit to the PAS and the anionic subsite in the CAS was worse. Compounds **7**–**9** with the diethylamine group were less active (0.92–1.23 μM) [39], because the aliphatic substituents (ethyl groups) could not provide as strong interactions as the aromatic phenyl ring in the benzylamine moiety. In the case of butyrylcholinesterase, due to the reduced peripheral binding site, interactions were weaker, and so, it led to inactivity against that enzyme.

### 2.5. Chemistry

**Figure 11:**
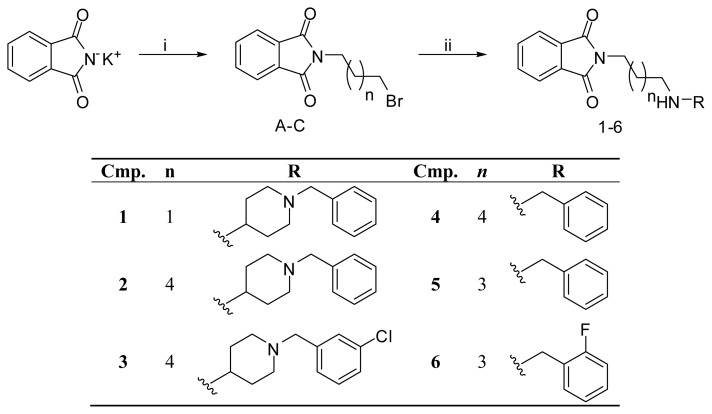
Reagents and conditions: i. dibromoalkane, tetra-*n*-butylammonium bromide (TBAB), MeCN, reflux, 20 h; ii. (**a**) 4-amino-1-benzylpiperidine, triethylamine (TEA), MeCN, reflux, 3 h (compound (cmp.) **1**,**2**); (**b**) 1-(3-chlorobenzyl)piperidin-4-amine, TEA, MeCN, microwave (MW), 100 °C, 45 min. (cmp. **3**); (**c**) benzylamine, K_2_CO_3_, MeCN, room temperature (RT), 48 h (cmp. **4**,**5**); **(d**) 2-fluorobenzylamine, K_2_CO_3_, MeCN, RT, 48 h (cmp. **6**).

The synthesis of the desired compounds was accomplished as illustrated in Figure 11. 2-Bromoalkylisoindole-1,3-diones (**A**–**C**) were prepared according to previously reported methods [39–41] in the reaction of potassium phthalimide and the appropriate dibromoalkane in acetonitrile with catalytic amount of tetra-*n*-butylammonium bromide (TBAB). They were used for preparation of final compounds **1**–**6** in the reaction with commercially available 4-amino-1-benzylpiperidine, benzylamine, 2-florobenzylamine and with 1-(3-chlorobenzyl)piperidin-4-amine, which was prepared as described below. The reaction conditions were adjusted to the substrates used, compounds **1**–**3** were prepared either by heating under a reflux or in a microwave synthesizer, whereas compounds **4**–**6** were obtained at room temperature.

1-(3-Chlorobenzyl)piperidin-4-amine [42] was prepared starting with 1-benzyl-4-aminopiperidine, whose amine group was tert-butyloxycarbonyl (BOC)-protected in the reaction with di-tert-butyl dicarbonate in tetrahydrofuran (THF). The obtained product was then debenzylated in methanol at atmospheric pressure of hydrogen and with a catalytical amount of 10% Pd/C and alkylated with 3-chlorobenzyl bromide in THF. The final product was obtained by BOC-deprotection in 10% HCl solution in methanol.

The final products were transformed into hydrochloride salts by adding to their solution isopropanol saturated with HCl.

### 2.6. Biological Assays

The activity against acetylcholinesterases from electric eel and butyrylcholinesterase from horse serum was determined in spectrophotometric Ellman’s assay. Tacrine and donepezil were used as reference compounds. The dose-response curve enabled us to calculate IC_50_ values and 95% confidence intervals. Both values for the activity of all compounds against cholinesterases are summarized in Table 5.

## 3. Experimental Section

### 3.1. Docking

The structures were prepared in the following way.

#### 3.1.1. Ligands

The 3D structures of all ligands were created by Corina on-line (Molecular Networks) [43] and saved as PDB files. Using Sybyl 8.0 (Tripos, St. Louis, MO, USA) [44], atom types were checked, protonation states were assigned, missing hydrogen atoms were added and Gasteiger charges were assigned. Finally, the compound structures were saved in mol2 format.

#### 3.1.2. Acetylcholinesterase

During the protein preparation (PDB: 1EVE [34]), all histidine residues were protonated at Nɛ, the hydrogen atoms were added, ligand molecules were removed, the binding site was defined as all amino acid residues within 10 Å from donepezil and three water molecules (1159, 1249, 1254) were included. Dockings were performed with Gold 4.1 [36]. A standard set of genetic algorithm with a population size of 100, a number of operations of 100,000 and clustering with a tolerance of 1 Å was applied. ChemScore was used for evaluation of the docking results. For each ligand, the final results involved 10 poses, arranged on the ranking list, according to the scoring function values.

#### 3.1.3. Butyrylcholinesterase

The enzyme structure (PDB: 1P0I [34]) was prepared for docking with Gold 4.1 [36]. Hydrogen atoms for the entire protein were added, protonating all histidine residues at Nɛ. Ligand molecules were removed, and the binding site was defined as the amino acid residues within 20 Å from the glycerol molecule, present in the active center of the enzyme. In the process of docking, water molecules that were present in the active center were taken into account, setting them as “toggle”, *i.e.*, the program decided whether to include them or not. A standard set of genetic algorithms with a population size of 100, a number of operations of 100,000 and clustering with a tolerance of 1 Å was applied, and the ChemScore function was used for scoring. As a result, 10 ligand poses, sorted by the scoring function value, were obtained.

### 3.2. Synthesis and Biological Assay

#### 3.2.1. General Information

##### 3.2.1.1. Chemistry

Microwave reactions were performed in a Discover LabMate from CEM Corporation. Column chromatography was performed on Merck silica gel 60 (63–200 μm). Analytical thin layer chromatography was done using aluminum sheets precoated with silica gel 60 F_254_. Analytical RPLC-MS was performed on Waters Acquity TQD with a mass spectrometer (Waters TQD, Milford, MA, USA) with detection by UV (DAD) using an Acquity UPLC BEH C_18_ column (1.7 μm, 2.1 × 100 mm). The CH_3_CN/H_2_O gradient with 0.1% HCOOH was used as the mobile phase at a flow rate of 0.3 mL/min. ^1^H NMR spectra were recorded on Varian Mercury 300 at 300 MHz. The chemical shifts for ^1^H NMR are referenced to TMS via residual solvent signals (^1^H, CDCl_3_ at 7.26 ppm, (CD_3_)_2_SO at 2.50 ppm). Elemental analyses were performed on a Vario EL III Elemental analyzer. All the compounds showed purity above 95%. The purity was determined by elemental analyses or on an analytical RPLC-MS on a Waters Acquity TQD using an Acquity UPLC BEH C18 column (1.7 μm, 2.1 × 100 mm) at 214 nm and 254 nm. The CH_3_CN/H_2_O gradient with 0.1% HCOOH was used as the mobile phase at a flow rate of 0.3 mL/min.

The following compounds: 2-(3-bromopropyl)isoindoline-1,3-dione (**A**) [40], 2-(5-bromopentyl)isoindoline-1,3-dione (**B**) [39], 2-(6-bromohexyl)isoindoline-1,3-dione (**C**) [41], 1-(3-chlorobenzyl)piperidin-4-amine [42], 2-(8-diethylaminooctyl)-isoindoline-1,3-dione (**7**) [39], 2-(7-diethylaminoheptyl)-isoindoline-1,3-dione (**8**) [39] and 2-(6-diethylaminohexyl)-isoindoline-1,3- dione (**9**) [39], were previously reported.

##### 3.2.1.2. Materials

All the reagents were purchased from commercial suppliers and were used without further purification. Tetrahydrofuran (THF) was distilled under nitrogen immediately before use; a mixture of sodium/benzophenone ketyl was used as a drying agent.

##### 3.2.1.3. Biological Activity

The activity against cholinesterases was determined in spectrophotometric Ellman’s assay [37].

#### 3.2.2. Experimental Details and Spectroscopic Data

2-(3-Bromopropyl)isoindoline-1,3-dione (A) [40]

^1^H NMR (300 MHz, CDCl_3_) δ ppm: 7.81–7.85 (m, 2H), 7.70–7.75 (m, 2H), 3.83 (t, *J =* 6.83 Hz, 2H), 3.41 (t, *J =* 6.75 Hz, 2H), 2.26 (q, *J =* 6.80 Hz, 2H).

2-(5-Bromopentyl)isoindoline-1,3-dione (B) [39]

^1^HNMR (CDCl_3_) δ ppm: 7.80–7.86 (m, 2H, Pht–*H*), 7.63–7.74 (m, 2H, Pht-*H*), 3.69 (t, *J =* 7.19 Hz, 2H, PhtC*H*_2_), 3.39 (t, *J =* 6.75 Hz, 2H, BrC*H*_2_), 1.85–1.95 (m, 2H, BrCH_2_C*H*_2_), 1.56–1.68 (m, 2H, PhtCH_2_C*H*_2_), 1.44–1.54 (m, 2H, Br(CH_2_)_2_C*H*_2_).

2-(6-Bromohexyl)isoindoline-1,3-dione (C) [41]

A mixture of potassium phthalimide (27.0 mmol, 5.00 g), 1,6-dibromohexane (67.5 mmol, 10.42 mL) and a catalytic amount of TBAB (1.0 mmol, 0.32 g) in acetonitrile (70.0 mL) was refluxed for 20 h. After the mixture was cooled down to room temperature, the solid salts were filtered off and the resulting filtrate was evaporated. The crude product was purified by column chromatography using *n*-hexane to wash out the access of 1,6-dibromohexane and *n*-hexane/EtOAc (7/3) for further purification. 6.76 g (81%) of a pure product was obtained. ^1^H NMR (300 MHz, CDCl_3_) δ ppm: 7.80–7.87 (m, 2H), 7.68–7.73 (m, 2H), 3.68 (t, *J* = 7.19 Hz, 2H), 3.39 (t, *J =* 6.79 Hz, 2H), 1.80–1.90 (m, 2H), 1.64–1.74 (m, 2H), 1.3–1.53 (m, 4H).

1-(3-Chlorobenzyl)piperidin-4-amine [42]

To a solution of 1-benzyl-4-aminopiperidine (70.0 mmol, 14.3 mL) and TEA (210.0 mmol, 29.3 mL) in 80.0 mL of THF cooled on an ice-bath, di-tert-butyl dicarbonate (77.0 mmol, 16.80 g) solution in 80.0 mL of THF was slowly added. The reaction mixture was warmed up to room temperature and left overnight. After the reaction was finished, the solvent was removed, and the obtained precipitate was dissolved in 250.0 mL of ethyl acetate and extracted with water (3 × 100.0 mL). The combined organic layers were dried over anhydrous Na_2_SO_4_ filtered and concentrated under vacuum to give 1-benzyl-4-(tert-butoxycarbonylamino)piperidine as a white solid 19.8 g (97%), MS: *m*/*z* 291 (M+H^+^).

To a solution of 1-benzyl-4-(tert-butoxycarbonylamino)piperidine (17.2 mmol, 5.00 g) in methanol (200.0 mL), 10% Pd/C (0.50 g) was added, and the mixture was hydrogenated at atmospheric pressure for 8 h. After this time, another portion of 10% Pd/C (0.50 g) was added, and the mixture was again left at atmospheric pressure of hydrogen for 8 hours; after that, the procedure was repeated again. After 24 h, the mixture was filtered through a Celite pad, concentrated under vacuum and purified by column chromatography in DCM/MeOH/TEA (85/10/5) to give 2.87 g (83%) of 4-(tert-butoxycarbonylamino)piperidine as a white solid. ^1^H NMR (300 MHz, CDCl_3_) δ ppm: 4.43–4.58 (m, 1H), 3.43–3.63 (m, 1H), 3.03–3.10 (m, 2H), 2.62–2.71 (m, 2H), 2.40 (s, 1H), 1.91–1.96 (m, 2H), 1.44 (s, 9H), 1.23–1.37 (m, 2H); MS: *m*/*z* 201 (M+H^+^).

To a solution of 4-(tert-butoxycarbonylamino)piperidine (10.0 mmol, 2.00 g) in 25.0 mL of anhydrous THF, 3-chlorobenzyl bromide (10.0 mmol, 1.32 mL) was added, and the mixture was refluxed for 24 h. After the reaction was finished, the solvent was evaporated and the crude product was purified by column chromatography on silica gel (DCM/MeOH, 95/5) to give 1.92 g (59%) of tert-butyl 1-(3-chlorobenzyl)piperidin-4-ylcarbamate as a white solid. ^1^H NMR (300 MHz, CDCl_3_) δ ppm: 7.32 (s, 1H), 7.15–7.25 (m, 3H), 4.38–4.50 (m, 1H), 3.45 (s, 3H), 2.76–2.80 (m, 2H), 2.05–2.14 (m, 2H), 1.85–1.97 (m, 2H), 1.37–1.51 (m, 11H); MS: *m*/*z* 325 (M+H^+^).

Tert-butyl-1-(3-chlorobenzyl)piperidin-4-ylcarbamate (11.3 mmol, 3.65 g) was dissolved in 100.0 mL 10% HCl in MeOH and was left stirring at room temperature for 24 h. After this time, the solvent was evaporated and the obtained oil was neutralized with 6 N NaOH and extracted with DCM and EtOAc (5 × 20 mL). The combined organic layers were dried over anhydrous Na_2_SO_4_, filtered and evaporated under vacuum to give the desired product as a brownish oil (0.92 g, 35%). ^1^H NMR (300 MHz, DMSO-d_6_) δ ppm: 11.30 (br. s., 1H), 8.50 (br. s., 2H), 7.78 (s, 1H), 7.40–7.64 (m, 3H), ), 4.26 (br. s., 2H), 3.15–3.35 (m, 3H), 2.97–3.08 (m, 2H), 1.89–2.23 (m, 4H); MS: *m*/*z* 225 (M+H^+^).

2-(3-(1-Benzylpiperidin-4-ylamino)propyl)isoindoline-1,3-dione (1)

A mixture of 2-(3-bromopropyl)isoindole-1,3-dione (5.0 mmol, 1.34 g), 4-amino-1-benzylpiperidine (5.0 mmol, 0.95 g) and TEA (6.0 mmol, 0.60 g) in 15.0 mL of acetonitrile was refluxed for 3 h. After cooling to room temperature, the expected product precipitated as a hydrobromide salt. It was collected by filtration, washed with MeCN and dried *in vacuo*. The obtained product was dissolved in 25.0 mL of water, and then, it was adjusted to basic pH with 25% NH_4_OH solution. Then, the product was extracted with dichloromethane (3 × 25.0 mL). The combined extracts were dried over anhydrous Na_2_SO_4_ to give 0.94 g (50%) of colorless oil. ^1^H NMR (300 MHz, CDCl_3_) δ ppm: 7.78–7.84 (m, 2H, Pht–*H*), 7.67–7.75 (m, 2H, Pht–*H*), 7.26–7.45 (m, 5H, Ar*H*), 3.84 (t, *J =* 6.31 Hz, 2H, PhtC*H*_2_), 3.68 (s, 2H, benzylC*H*_2_), 2.87–3.15 (m, 5H: 3H, Ppd, 2H, NHC*H*_2_), 2.19–2.38 (m, 6H: 4H, Ppd, 2H PhtCH_2_C*H*_2_), 1.88–1.99 (m, 2H, Ppd), NH signal not detected. MS: *m*/*z* 378 (M+H^+^). Analysis calculated (Anal. calcd.) for C_23_H_29_N_3_O_2_Cl_2_: (%) C, 61.33; H, 6.49; N, 9.33. Found: C, 61.06; H, 6.35; N, 8.85.

2-(6-(1-Benzylpiperidin-4-ylamino)hexyl)isoindoline-1,3-dione (2)

A mixture of 2-(6-bromohexyl)isoindole-1,3-dione (5.0 mmol, 1.48 g), 4-amino-1-benzylpiperidine (5.0 mmol, 0.95 g) and TEA (6.0 mmol, 0.60 g) in 15.0 mL of acetonitrile was refluxed for 3 h. After cooling, the expected product precipitated as a hydrobromide salt. It was collected by filtration, washed with MeCN and dried *in vacuo*. The obtained product was dissolved in 25.0 mL of water and then was adjusted to basic pH with 25% NH_4_OH solution. Then, the product was extracted with dichloromethane (3 × 25.0 mL). The combined extracts were dried over anhydrous Na_2_SO_4_ to give 1.40 g (67%) of colorless oil. ^1^H NMR (300 MHz, CDCl_3_) δ ppm: 7.78–7.84 (m, 2H, Pht–*H*), 7.66–7.71 (m, 2H, Pht–*H*), 7.22–7.30 (m, 5H, Ar*H*), 3.63 (t, *J =* 7.04 Hz, 2H, PhtC*H*_2_), 3.52 (s, 2H, benzylC*H*_2_), 2.82–3.04 (m, 5H: 2H, NHC*H*_2_, 3H, Ppd), 1.79–2.19 (m, 8H: 6H, Ppd, 2H, NHCH_2_C*H*_2_), 1.59–1.73 (m, 2H, PhtCH_2_C*H*_2_), 1.28–1.46 (m, 4H, Pht(CH_2_)_2_(C*H*_2_)_2_), NH signal not detected. MS: *m*/*z* 420 (M+H^+^). Anal. calcd. for C_26_H_35_N_3_O_2_Cl_2_ (%) C, 63.41; H, 7.16; N, 8.53. Found: C, 63.23; H, 7.18; N, 8.50.

2-(6-(1-(3-Chlorobenzyl)piperidin-4-ylamino)hexyl)isoindoline-1,3-dione (3)

A 2–5 mL microwave-transparent process vial was charged with 2-(6-bromohexyl)isoindoline-1,3- dione (0.35 mmol, 108.6 mg), 1-(3-chlorobenzyl)piperidin-4-amine (0.35 mmol, 78.7 mg), TEA (0.42 mmol, 58.6 μL) and acetonitrile (2.0 mL). The mixture was exposed to microwave heating for 45 min at 100 °C. After the mixture was cooled, the solvent was evaporated and the crude product was purified by column chromatography in DCM/MeOH (9/1) to give 0.11 g (70%) of a white solid. ^1^H NMR (300 MHz, CDCl_3_) δ ppm: 7.79–7.85 (m, 2H, Pht–*H*), 7.68–7.74 (m, 2H, Pht–*H*), 7.32 (s, 1H, Ar*H*), 7.20–7.25 (m, 3H, Ar*H*), 3.65 (t, *J* = 7.05 Hz, 2H, PhtC*H*_2_), 3.50 (s, 2H, benzylC*H*_2_), 3.03–3.15 (m, 1H, Ppd), 2.89–3.01 (m, 4H: 2H, NHC*H*_2_, 2H, Ppd), 2.18–2.22 (m, 2H, Ppd), 1.88–2.13 (m, 6H: 4H Ppd, 2H NHCH_2_C*H*_2_), 1.63–1.73 (m, 2H, PhtCH_2_C*H*_2_), 1.30–1.50 (m, 4H, Pht(CH_2_)_2_(C*H*_2_)_2_); MS: *m*/*z* 454 (M+H^+^).

2-(6-(Benzylamino)hexyl)isoindoline-1,3-dione (4)

A mixture of 2-(6-bromohexyl)isoindole-1,3-dione (0.50 mmol, 0.16 g), benzylamine (1.50 mmol, 0.16 g) and K_2_CO_3_ (1.50 mmol, 0.21 g) was stirred in 20.0 mL acetonitrile for 48 h. After the reaction was finished, the solvent was evaporated and the obtained residue was dissolved in 20.0 mL water and extracted with chloroform (4 × 10.0 mL). The organic layer was dried over Na_2_SO_4_ and evaporated. The crude product was purified by column chromatography in CHCl_3_/MeOH (98/2, 95/5) to give 78.0 mg (46%) of a yellow oil. ^1^ H NMR (300 MHz, CDCl_3_) δ ppm: 7.84–7.81 (m, 2H, Pht), 7.73–7.68 (m, 2H, Pht), 7.45–7.26 (m, 5H, Ph), 3.89 (s, 2H, *CH_2_*Ph), 3.67 (t, *J* = 7.2 Hz, 2H, Pht*CH_2_*), 2.68 (t, *J* = 7.5 Hz, 2H, (CH_2_)_5_*CH_2_*NH), 1.71–1.60 (m, 2H, PhtCH_2_*CH_2_*), 1.37–1.25 (m, 6H, Pht(CH_2_)_2_*(CH_2_)_3_*), NH signal not detected; MS: *m*/*z* 337 (M+H^+^).

2-(5-(Benzylamino)pentyl)isoindoline-1,3-dione (5)

A mixture of 2-(5-bromopentyl)isoindole-1,3-dione (0.50 mmol, 0.15 g), benzylamine (1.50 mmol, 0.16 g) and K_2_CO_3_ ( 1.50 mmol, 0.21 g) was stirred in 20.0 mL acetonitrile for 48 h at room temperature. After the reaction was finished, the solvent was evaporated and the obtained residue was dissolved in 20.0 mL water and extracted with chloroform (4 × 10.0 mL). The organic layer was dried over Na_2_SO_4_ and evaporated. The crude product was purified by column chromatography in CHCl_3_/MeOH (98/2, 95/5) to give 80.0 mg (50%) of a yellow oil. The final product was obtained in the form of hydrochloride salt. ^1^ H NMR (300 MHz, (CD_3_)_2_SO) δ ppm: 9.00 (s, 2H, NH_2_^+^), 7.87–7.81 (m, 4H, Pht), 7.51–7.49 (m, 2H, Ph), 7.41–7.39 (m, 3H, Ph), 4.10–4.06 (t, *J* = 5.8 Hz, 2H, NH_2_^+^*CH_2_*Ph), 3.55 (t, *J* = 7.0 Hz, 2H, Pht*CH_2_*), 2.89–2.80 (m, 2H, (CH_2_)_4_*CH_2_*NH_2_^+^), 1.67–1.62 (m, 2H, *CH_2_*CH_2_NH_2_^+^), 1.61–1.55 (m, 2H, PhtCH_2_*CH_2_*), 1.31–1.27 (m, 2H, (CH_2_)_2_*CH_2_*(CH_2_)_2_); MS: *m*/*z* 323 (M+H^+^).

2-(5-(2-Flourobenzylamino)pentyl)isoindoline-1,3-dione (6)

A mixture of 2-(5-bromopentyl)isoindole-1,3-dione (0.50 mmol, 0.15 g), 2-florobenzylamine (1.50 mmol, 0.19 g) and K_2_CO_3_ (1.50 mmol, 0.21 g) was stirred in 20.0 mL acetonitrile for 48 h. After the reaction was finished, the solvent was evaporated and the obtained residue was dissolved in 20.0 mL water and extracted with chloroform (4 × 10.0 mL). The organic layer was dried over Na_2_SO_4_ and evaporated. The crude product was purified by column chromatography in CHCl_3_/MeOH (98/2, 95/5) to give 80.0 mg (47%) of a yellow oil. ^1^ H NMR (300 MHz, CDCl_3_) δ ppm: 7.88–7.79 (m, 2H, Pht), 7.73–7.67 (m, 2H, Pht), 7.36–7.28 (m, 1H, Ph), 7.23–7.18 (m, 1H, Ph), 7.11–6.98 (m, 2H, Ph), 3.79 (s, 2H, *CH_2_*phenyl), 3.67 (t, *J* = 7.2 Hz, 2H, Pht*CH_2_*), 2.61 (t, *J* = 7.2 Hz, 2H, (CH_2_)_4_*CH_2_*NH), 1.73–1.66 (m, 2H, PhtCH_2_*CH_2_*), 1.61–1.51 (m, 2H,*CH_2_*CH_2_NH), 1.43–1.32 (m, 2H, (CH_2_)_2_*CH_2_*(CH_2_)_2_), NH signal not detected; MS: *m*/*z* 341 (M+H^+^).

#### 3.2.3. Ellman’s Assay—AChE/BuChE Inhibition Activity

Reagents and chemicals—acetylthiocholine iodide, butyrylthiocholine iodide, AChE from *Electrophorus electricus* (425.96 U/mg solid), BuChE from horse serum (2.5 units/1 mL) and DNTB—were purchased from Sigma–Aldrich. All assays were performed using the Perkin Elmer Lambda 12 device with detection at 412 nm. The reaction took place in 100 mM phosphate buffer, pH 8.0, containing 0.25 units of AChE or BuChE, 0.3 mmol 5,5′-dithio-bis-(2-nitrobenzoic) acid (DTNB) and 0.45 mmol acetylthiocholine or butyrylthiocholine as substrates. The tested compounds were incubated with the enzyme for 5 min at 25 °C prior to starting the reaction by adding the substrate. Enzymatic activity was determined by measuring absorbance over a period of 5 min. Two kinds of assays were conducted: the peak activity of the enzyme was determined by using a blank sample, followed by reference measurements (for tacrine and donepezil, see table below) and measurements for the synthesized compounds. Data from concentration-inhibition experiments were integrated through nonlinear regression analysis using the GraphPad Prism program (GraphPad Prism Software Inc. 2005, La Jolla, CA, USA), producing estimates of IC_50_.

## 4. Conclusions

Molecular modeling studies allowed us to propose new structures as potential cholinesterase inhibitors, derived from a previously developed library or from structural fragments.

According to the modeling studies, compound **1** and **2** bind through the main and peripheral AChE active site due to π–π stacking interactions with Trp84 and Trp279 residues. We assume that these interactions are responsible for the inhibitory activity of these compounds against acetylcholinesterase and against β-amyloid aggregation. On the basis of these structures, new derivatives with potentially enhanced activity were designed. The design included changes of the heterocyclic system, the length of the linker and the introduction of substituents into the benzyl moiety. As heterocyclic systems, phthalimide, indole, tetrahydroisoquinoline, tetrahydroquinoline and isoindoline were sequentially tested computationally. The most preferred fragment for the short linker (three carbon atoms) was phthalimide. The analysis of the influence of substituents in the benzyl moiety on potential activity involved fluorine, chlorine, hydroxy, methoxy and trifluoromethyl groups and dimethyl or ethyl carbamate. For all substituents, it was examined which position in the phenyl ring was most preferred. It was found that *p*-hydroxy, *m*-chloro and *o*-fluoro derivatives were the highest rated. For the six carbon linker, different heterocyclic rings obtained similar score, but in the case of an eight-carbon linker, three moieties were distinguished: isoindoline, tetrahydroquinoline and indole. In every case, *p*-hydroxy, *m*-chloro and *o*-fluoro derivatives were better than unsubstituted compounds. Derivative **3** (*m*-chloro analogue of compound **2**) was obtained, and it was an inhibitor of both cholinesterases with improved activity (AChE: 44.0 μM, BuChE: 7.66 μM).

These results, the fragment-based design technique and literature data were used for further design of potential inhibitors of cholinesterases. In this way, novel compounds with different amine moieties (benzylamine, *o*-fluorobenzylamine or diethylamine) were designed and preliminary studies on synthesis and biological evaluation were made. The obtained and currently described [39] results confirmed that the investigated compounds **4**–**9** were selective inhibitors of AChE with IC_50_ values in the range 0.087–1.23 μM. They were more potent inhibitors than parent compounds from our library, and some of them displayed beta-amyloid anti-aggregating activity, which leads to the assumption that they might be dual binding site inhibitors. It is worthwhile to note that the IC_50_ value against acetylcholinesterase for the most active compound, **6** (87 nM), was in the same range as for donepezil (31 nM).

Summing up, the described structure-based search methodology led to the discovery of cholinesterase inhibitors with activities improved from the micromolar to the mid-nanomolar range. This approach could represent a useful tool for the discovery of novel cholinesterases inhibitors potentially endowed with additional properties.

## Figures and Tables

**Table 1:** Comparison of the TcAChE active site from different crystal complexes from the PDB: root-mean-square deviation (rmsd) values in Å for all atoms: the amino acids included in the comparison are presented in Figure 2.

PDB	1ACJ	1ACL	1AX9	1DX6	1EVE	1GQR	1VOT	2ACE	2BAG	2CKM	2VQ6
1ACJ	0	1.062	0.784	0.915	0.907	1.030	0.803	0.830	0.907	1.059	0.751
1ACL		0	0.728	0.732	0.691	0.850	0.706	0.757	0.663	1.144	0.852
1AX9			0	0.722	0.714	0.760	0.274	0.617	0.692	0.878	0.536
1DX6				0	0.366	0.711	0.720	0.511	0.318	0.934	0.614
1EVE					0	0.634	0.710	0.488	0.236	0.975	0.641
1GQR						0	0.781	0.625	0.579	1.040	0.743
1VOT							0	0.594	0.692	0.915	0.581
2ACE								0	0.443	0810	0.573
2BAG									0	0.958	0.627
2CKM										0	0.844
2VQ6											0

Complexes with inhibitors: 1ACJ—tacrine, 1ACL—decamethonium, 1AX9—edrophonium, 1DX6—galantamine, 1EVE—donepezil, 1GQR—rivastigmine, 1VOT—huperzine A, 2ACE—native, 2BAG—ganstigmine, 2CKM—bis-7-tacrine, 2VQ6—pralidoxime; green color means the pair with the smallest differences; red color means the pair with the largest differences.

**Table 2:** Optimal settings of Gold for redockings of seven reference inhibitors.

Complex	Binding site-radius (Å)	Water molecules	Scoring function	Value of scoring function	rmsd (Å)
1EVE	10	1159, 1249, 1254 (toggle *)	ChemScore	49.48	0.8
1ACJ	12	616, 634, 643 (toggle)	GoldScore	67.95	0.4
2CKM	10	None	GoldScore	80.72	1.3
1DX6	12	None	GoldScore	59.34	0.6
1ACL	10	None	GoldScore	40.45	1.6
1AX9	12	None	ChemScore	26.36	1.4
1VOT	12	619, 680 (toggle)	ChemScore	41.60	0.9

* The program decides at the step of docking if the water molecule is engaged in ligand binding.

**Table 3:** Results of cross-dockings to seven analyzed crystal structures of AChE. Assessment with rmsd values (blue: good pose; green: close pose; yellow: pose with errors; pink: bad pose).

	1EVE	1ACJ	2CKM	1DX6	1ACL	1AX9	1VOT
donepezil	0.8	9.3	10.3	2.6	3.4	2.9	9.6
tacrine	7.6	0.4	2.9	6.0	5.3	9.6	6.1
bis-7-tacrine	4.3	5.7	1.3	5.1	5.1	4.8	5.0
galantamine	1.1	3.7	0.8	0.6	6.2	8.7	4.9
decamethonium	1.4	3.2	2.3	2.5	1.6	3.2	2.4
edrophonium	4.5	2.8	2.2	2.3	2.4	1.4	2.0
huperzine A	5.0	5.0	3.5	3.6	5.0	3.7	0.9

**Table 4:** Assessment of the investigated compounds as potential AChE inhibitors.

Compound	ChemScore AChE	IC_50_ AChE (nM)
4	45.29	330
5	41.83	170
6	41.91	87.2
7	40.54	920
8	41.35	1070
9	38.42	1230
donepezil	49.48	31.2

IC_50_ values were determined in Ellman’s assay [37,39]. The reference compound, donepezil, was placed in the table for comparison.

**Table 5:** Anticholinesterases activity of designed compounds.

Compound	AChE	AChE	BuChE	BuChE

	IC_50_ (μM)	95% CI (μM)	IC_50_ (μM)	95% CI (μM)
**1**	18.2	14.2–23.3	22.8	13.1–39.8
**2**	53.7	40.6–71.1	24.5	15.9–37.7
**3**	44.0	40.2–48.3	7.66	5.92–9.91
**4**	0.330	0.313–0.348	n.a.	n.a.
**5**	0.170	0.153–0.188	n.a.	n.a.
**6**	0.0872	0.0777–0.0980	n.a.	n.a.
**7**	0.922	0.772–1.100	67.5	59.9–76.1
**8**	1.07	0.88–1.30	n.a	n.a.
**9**	1.23	1.06–1.42	n.a	n.a.
**Donepezil**	0.0312	0.0274–0.0355	2.84	2.49–3.24
**Tacrine**	0.0503	0.0465–0.0543	0.00536	0.00439–0.00655

n.a.: not active; 95% CI: 95% confidence interval.
